# REverSe TRanscrIptase chain termination (RESTRICT) for selective measurement of nucleotide analogs used in HIV care and prevention

**DOI:** 10.1002/btm2.10369

**Published:** 2022-07-14

**Authors:** Ayokunle O. Olanrewaju, Benjamin P. Sullivan, Alicia H. Gim, Cosette A. Craig, Derin Sevenler, Andrew T. Bender, Paul K. Drain, Jonathan D. Posner

**Affiliations:** ^1^ Department of Mechanical Engineering University of Washington Seattle Washington USA; ^2^ Department of Bioengineering University of Washington Seattle Washington USA; ^3^ Department of Chemical Engineering University of Washington Seattle Washington USA; ^4^ Center for Engineering in Medicine and Surgery Massachusetts General Hospital Boston Massachusetts USA; ^5^ Department of Epidemiology University of Washington Seattle Washington USA; ^6^ Department of Global Health University of Washington Seattle Washington USA; ^7^ Department of Medicine University of Washington Seattle Washington USA; ^8^ Department of Family Medicine University of Washington Seattle Washington USA

**Keywords:** adherence monitoring, enzymatic assay, nucleotide analog, nucleotide reverse transcriptase inhibitor, reverse transcriptase inhibitor, tenofovir diphosphate, therapeutic drug monitoring

## Abstract

Sufficient drug concentrations are required for efficacy of antiretroviral drugs used in HIV care and prevention. Measurement of nucleotide analogs, included in most HIV medication regimens, enables monitoring of short‐ and long‐term adherence and the risk of treatment failure. The REverSe TRanscrIptase Chain Termination (RESTRICT) assay rapidly infers the concentration of intracellular nucleotide analogs based on the inhibition of DNA synthesis by HIV reverse transcriptase enzyme. Here, we introduce a probabilistic model for RESTRICT and demonstrate selective measurement of multiple nucleotide analogs using DNA templates designed according to the chemical structure of each drug. We measure clinically relevant concentrations of tenofovir diphosphate, emtricitabine triphosphate, lamivudine triphosphate, and azidothymidine triphosphate with agreement between experiment and theory. RESTRICT represents a new class of activity‐based assays for therapeutic drug monitoring in HIV care and could be extended to other diseases treated with nucleotide analogs.

## 1 INTRODUCTION

The development and availability of antiretroviral drugs has decreased the number of deaths related to Human Immunodeficiency Virus (HIV) by 40% over the past decade.^1^ However, sufficient drug concentrations are required to obtain the individual and population‐level benefits of antiretroviral therapy (ART) and pre‐exposure prophylaxis (PrEP) regimens for HIV treatment and prevention, respectively.^2^, ^3^, ^4^, ^5^ In several PrEP trials and implementation studies, concentrations of antiretroviral medications in blood were correlated with PrEP efficacy.^6^ Similarly, viral suppression was associated with antiretroviral concentration among people living with HIV receiving ART.^4^ Regular monitoring of antiretroviral drug levels and provision of appropriate counseling/feedback could promote ART and PrEP adherence, increase antiretroviral drug concentrations, and improve HIV care.^3^

Nucleotide analogs are a suitable target for antiretroviral drug monitoring because of their inclusion in most ART and PrEP regimens^7^ and their favorable pharmacokinetics.^8^ Nucleotide analogs terminate DNA synthesis by HIV reverse transcriptase (RT) and prevent HIV replication. Tenofovir diphosphate (TFV‐DP), a deoxyadenosine analog, is used in over 80% of ART regimens and in all approved oral PrEP regimens.^7^, ^9^ TFV‐DP has a 17‐day half‐life in red blood cells (RBCs) and accumulates 25‐fold at steady state compared to the start of drug ingestion, which enables monitoring of long‐term medication adherence over a 1 to 3 month period.^10^, ^11^, ^12^ Meanwhile, emtricitabine triphosphate (FTC‐TP), a deoxycytidine analog, is also included in several ART regimens and in all approved oral PrEP regimens. FTC‐TP has a 35‐h half‐life in RBCs and provides information about recent (1‐week) adherence.^13^ Measurement of TFV‐DP and FTC‐TP concentrations can provide information about short‐term and long‐term adherence and enable investigation of their implications in clinical practice and behavioral science studies.^4^, ^10^, ^11^, ^13^, ^14^, ^15^, ^16^

Liquid chromatography tandem mass spectrometry (LC‐MS/MS) is the gold standard for measuring nucleotide analogs,^10^, ^11^, ^17^, ^18^ and was used in directly observed therapy trials to determine TFV‐DP concentrations corresponding to low (2 doses/week), intermediate (4 doses/week), and high (7 doses/week) PrEP adherence.^10^, ^11^ LC‐MS/MS was used to establish FTC‐TP thresholds corresponding to recent medication ingestion.^10^, ^11^, ^13^ Although LC‐MS/MS provides accurate and quantitative information about TFV‐DP and FTC‐TP concentrations, it requires complex sample preparation, highly trained operators, and resource intensive equipment that limit its use to centralized and highly capable laboratories. This hinders routine measurement of antiretroviral concentrations and could delay counseling and interventions in behavioral science studies.^3^, ^19^ There are ongoing efforts to miniaturize LC‐MS/MS instruments and to integrate the required sample preparation steps into miniaturized cartridges.^20^ ^,^ ^21^, ^22^ However, the per‐unit cost and complexity of current miniaturized LC‐MS/MS systems still exceeds requirements in low and middle‐income settings.

A rapid and inexpensive test that measures nucleotide analogs indicative of long‐ and short‐term adherence in point‐of‐care (POC) settings like a doctor's office, patient's home, or event setting could be beneficial in monitoring and improving ART and PrEP outcomes.^3^, ^23^ Immunoassays and lateral flow assays for measuring antiretroviral drugs were recently developed.^24^, ^25^, ^26^, ^27^ Most immunoassays for HIV adherence measurement target tenofovir (TFV), a precursor of TFV‐DP that is present in plasma and urine.^12^ TFV has a 15‐h half‐life in plasma and indicates recent ingestion of medication within the prior 48 h. The short half‐life of TFV makes its measurement susceptible to conflating recent medication ingestion (≤1 week) with long‐term (1–3 months) adherence. We recently developed an immunoassay for measuring clinically relevant concentrations of both TFV and TFV‐DP^28^; however, additional sample preparation is required to separate RBCs from plasma because the antibodies used could not distinguish between TFV and TFV‐DP. There is still a strong need for assays that can selectively measure biomarkers of short‐ and long‐term in POC settings.^3^, ^29^

**FIGURE 1:**
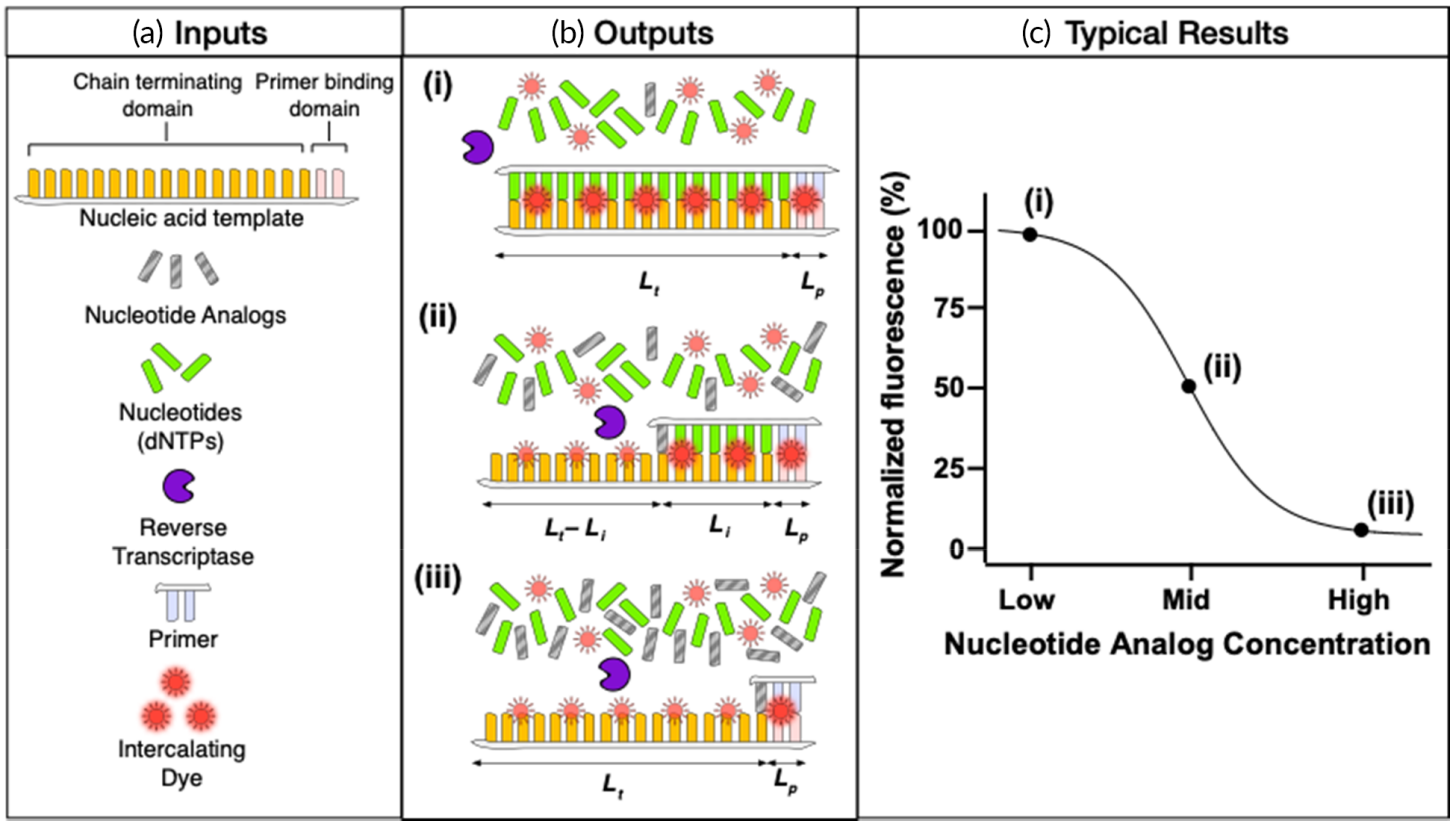
Summary of inputs and outputs of RESTRICT assay. (a) RESTRICT uses nucleic acid templates, nucleotides, recombinant HIV‐1 reverse transcriptase, primers, and intercalating dyes to measure the concentration of nucleotide analogs. The nucleic acid templates have primer‐binding domains to initiate DNA synthesis and chain terminating domains designed to bind specific nucleotide analogs. (b) The key contributions to fluorescence output of the RESTRICT assay come from (i) full‐length dsDNA products, (ii) fragment dsDNA products, and (iii) unpolymerized ssDNA template. (c) Typical sigmoidal fluorescence versus concentration profile obtained from RESTRICT assays illustrating the high, intermediate, and low fluorescence obtained from the different assay end products

We recently developed the REverSe TranscrIptase Chain Termination (RESTRICT) assay for detection of nucleotide analogs based on their enzyme inhibition activity.^30^ Unlike immunoassays, RESTRICT directly measures the pharmacologic activity of the analyte. RESTRICT uses DNA templates, nucleotides, recombinant HIV‐1 reverse transcriptase, primers, and intercalating dyes to infer the concentration of nucleotide analogs based on the inhibition of DNA synthesis by recombinant HIV RT (Figure 1). DNA chain termination occurs at high nucleotide analog concentration resulting in low DNA synthesis and low fluorescence reported by the intercalating dye. Meanwhile at low nucleotide analog concentration, there is extensive DNA synthesis and high fluorescence from the intercalating dye.

We use dilution in water as a simple and user‐friendly sample preparation mechanism to release intracellular TFV‐DP from RBCs and decrease non‐specific inhibition by blood matrix components.^31^ In a pilot clinical evaluation, RESTRICT measured clinically relevant TFV‐DP concentrations in whole blood and identified participants with TFV‐DP concentrations corresponding to adequate adherence (≥700 fmol per 3 mm dried blood spot [DBS] punch) among PrEP clients. RESTRICT represents a new class of activity‐based diagnostic assays with potential for rapid, POC measurement of antiretroviral drugs used in HIV treatment and prevention.

In this article, we demonstrate that RESTRICT can selectively measure multiple nucleotide analogs based on their chemical structure. Guided by a probabilistic model of RT inhibition, we investigate the impact of assay parameters including nucleotide analog affinity, nucleotide concentration, template length, and template sequence on RESTRICT. We demonstrate selective detection of biomarkers of either long‐term or short‐term adherence without cross‐reactivity using DNA templates that are rich in the endogenous nucleotide that the drug mimics.

## 2 THEORY

### 2.1 Overview and key assumptions

We present a probabilistic reaction equilibrium model of RESTRICT. We assume that incorporation of dNTP or nucleotide analog occurs as a random probabilistic event that depends primarily on their relative concentrations. For simplicity, we ignore time‐dependent changes in reagent concentration and assume that dNTP and nucleotide analog are available in excess for DNA chain elongation or termination. This model recapitulates experimental results and allows investigation of the role of assay parameters such as nucleotide analog affinity, dNTP concentration, template length, and template sequence on RESTRICT assay performance.

Nucleic acid templates used in the RESTRICT assay consist of a primer binding domain and a chain terminating domain (Figure 1a). The primer binding domain is located on the 3′ end of the template and guides primer initiation of DNA synthesis. The chain terminating domain is located directly downstream of the primer binding domain and is designed to enable preferential insertion of specific nucleotide analogs. For example, an assay designed to detect TFV‐DP, a deoxyadenosine analog, will use a DNA template rich in thymidine (T) bases to provide ample opportunity for TFV‐DP insertion. Although the ideal chain terminating domain for TFV‐DP detection is a homopolymeric template consisting of a string of T's, in practice, HIV RT exhibits template preferences and does not efficiently polymerize Poly(dT) templates,^32^, ^33^ so the template sequence must be optimized.

There are several contributions to the output fluorescence signal. The largest signal fraction originates from the interaction between intercalating fluorescent dyes and double stranded DNA (dsDNA) products. There are smaller magnitude contributions from unpolymerized single stranded DNA (ssDNA) templates and primers. Intercalating dyes provide significantly greater fluorescence when bound to dsDNA; however, they also produce a measurable fluorescence signal when bound to ssDNA (Figure 1b). For example, PicoGreen™ used in our experiments, provides 11 times greater fluorescence when bound to dsDNA compared to ssDNA.^34^, ^35^ The theoretical model also accounts for fluorescence from unpolymerized ssDNA. In the sections below, we provide calculations of the fluorescence contributions of different assay end products.

### 2.2 Full‐length dsDNA products

For a nucleic‐acid template with chain terminating domain length, $L_{t}$, and n bases complementary to the target nucleotide analog ($n\leq L_{t}$), the formation of full‐length dsDNA requires n consecutive dNTP insertion events. Assuming these are all independent dNTP insertion events in the presence of excess dNTP and nucleotide analog concentration, the probability of full‐length dsDNA formation can be expressed as,

(1) $$P_{\text{dNTP},n}={\left(\frac{\left[\text{dNTP}\right]}{\left[\text{dNTP}\right]+K_{\mathrm{aff}}\cdot \left[\mathrm{NA}\right]}\right)}^{n}$$

where $\left[\mathrm{NA}\right]$ is nucleotide analog concentration, $\left[\text{dNTP}\right]$ is nucleotide concentration, and $K_{\mathrm{aff}}$ is the relative affinity of RT enzyme for the nucleotide analog compared to dNTP.

The fluorescence from full‐length dsDNA products, $F_{\text{full}}$, depends on the length of the DNA template and the fluorescence properties of the intercalating dye and can be expressed as,

(2) $$F_{\text{full}}=C_{t}\cdot K_{\mathrm{dye}}\cdot L_{t}\cdot P_{\text{dNTP},n}$$

where $K_{\mathrm{dye}}$ is the fluorescence per double‐stranded base‐pair per unit concentration of the intercalating dye, and $C_{t}$ is the concentration of DNA template.

### 2.3 Fragment dsDNA products

There is a distribution of dsDNA fragments sizes during RESTRICT assays as previously observed in experimental studies of RT activity.^32^ Partial‐length dsDNA fragments contribute to the total fluorescence in the RESTRICT assay (Figure 1b). Like full‐length DNA, fluorescence from dsDNA fragments depends on the probability of formation of a dsDNA fragment and the length of the DNA fragment. For a given pair of nucleotide and nucleotide analog concentrations, we can calculate the probability of chain termination at any of the n possible insertion sites for nucleotide analogs in the DNA template. The probability of formation of a dsDNA fragment is,

(3) $$P_{\text{frag},i}=P_{\text{dNTP},i-1}\cdot P_{\mathrm{NA}}$$

where the index i≥1 counts the bases where it is possible to insert nucleotide analog and the maximum possible value of i is the total number of bases in the template, or simply n. $P_{\text{dNTP},i-1}$ is the probability that dNTP molecules were incorporated in the nucleic acid template at all bases preceding the base i where nucleotide analog was inserted and is calculated using Equation (1). $P_{\mathrm{NA}}$ is the probability of a single nucleotide analog insertion event and can be expressed as,

(4) $$P_{\mathrm{NA}}=1-P_{\text{dNTP},n=1}=\left(\frac{K_{\mathrm{aff}}\cdot \left[\mathrm{NA}\right]}{\left[\text{dNTP}\right]+K_{\mathrm{aff}}\cdot \left[\mathrm{NA}\right]}\right)$$

To determine the total fluorescence contribution from dsDNA fragments, we calculate the sum of fluorescence from all the dsDNA fragments. Adapting Equation (3) and summing the fluorescence from individual dsDNA fragments, the fluorescence from dsDNA fragments can be expressed as,

(5) $$F_{\text{dsDNA}-\text{frag}}=C_{t}\cdot K_{\mathrm{dye}}\cdot \sum_{i=1}^{n-1}\left(P_{\text{dNTP},i-1}\cdot P_{\mathrm{NA}}\cdot L_{i}\right)$$

where each termination site i and the resulting dsDNA fragment of length, $L_{i}$, is known since we know the exact sequence of DNA templates used in the assay.

### 2.4 Unpolymerized ssDNA

We also account for the background fluorescence contribution of intercalating dye interacting with unpolymerized nucleic acid template. At high nucleotide analog concentrations, very little (if any) dsDNA is formed and most of the fluorescence output comes from unpolymerized ssDNA template (Figure 1b). In RESTRICT, this background varies with nucleotide analog concentration. Each dsDNA fragment has a corresponding ssDNA fragment with length equal to the difference between the total length of the nucleic acid template and the dsDNA fragment (Figure 1b). Thus, the background fluorescence from the ssDNA fragments can be calculated as,

(6) $$F_{\text{ssDNA}-\text{frag}}=C_{t}\cdot \frac{K_{\mathrm{dye}}}{D}\cdot \sum_{i=1}^{n-1}\left(P_{\text{dNTP},i-1}\cdot P_{\mathrm{NA}}\cdot \left[L_{t}-L_{i}\right]\right)$$

where $\left[L_{t}-L_{i}\right]$ is the length of the unpolymerized single‐stranded portion of a fragment product that was terminated at base i, and the factor D is the fold reduction in fluorescence when intercalating dye is bound to ssDNA rather than dsDNA (D=11 for PicoGreen™)^34^, ^35^

**Contributions of bound and unbound primer:** DNA templates used in the RESTRICT assay all had a common 20 nt primer binding domain, while the chain terminating domain varied in sequence and length from 45 to 180 nt. Given that the primer binding domain constitutes up to 31% of the total template length, it is important to account for fluorescence that arises from the primer binding domain. Assuming the reaction goes to completion with all available templates bound to a primer, the fluorescence from dsDNA due to bound primers can be calculated as,

(7) $$F_{\text{bound}-\text{primer}}=C_{\text{template}}\cdot K_{\mathrm{dye}}\cdot L_{\text{primer}}$$

where $C_{\text{template}}$ is the template concentration and $L_{\text{primer}}$ is the primer length.

There is also background fluorescence from unbound primer in solution. To ensure that all templates bound to a primer, we used excess primer in the RESTRICT assay (10 times the template concentration). The fluorescence due to unbound ssDNA primer can be calculated as,

(8) $$F_{\text{free}-\text{primer}}=\left(C_{\text{primer}}-C_{t}\right)\cdot \frac{K_{\mathrm{dye}}}{D}\cdot L_{\text{primer}}$$

where $\left(C_{\text{primer}}-C_{t}\right)$ is the difference between primer and template concentration, and the factor D is the fold reduction in fluorescence when intercalating dye is bound to ssDNA rather than dsDNA (*D* = 11 for PicoGreen).^34^, ^35^

### 2.5 Total fluorescence from RESTRICT assay products

We can calculate the total fluorescence, $F_{\text{total}}$, at the end of the RESTRICT assay by combining Equations ((1), (2), (3), (4), (5), (6), (7), (8)),

(9) $$F_{\text{total}}=F_{\text{full}}+F_{\text{dsDNA}-\text{frag}}+F_{\text{ssDNA}-\text{frag}}+F_{\text{bound}-\text{primer}}+F_{\text{free}-\text{primer}}$$

Using this, we can estimate RESTRICT assay performance as we vary assay parameters such as nucleotide concentration, template length, and template sequence.

## 3 EXPERIMENTAL SECTION

### 3.1 RESTRICT assay reagents and workflow

RESTRICT reactions were carried out in buffer containing 60 mM Tris (77‐86‐1, Sigma, St. Louis, MO), 30 mM KCl (7447‐40‐7, Sigma, St. Louis, MO), 8 mM MgCl_2_ (7786‐30‐3, Sigma, St. Louis, MO), and 10 mM dithiothreitol (20‐265, Sigma, St. Louis, MO) buffered to pH 8.0 using HCl (7647‐01‐0, Acros Organics, Fair Lawn, NJ). Recombinant RT enzyme was obtained from Worthington Biochemical Corporation (LS05006, Lakewood, NJ). The nucleotide analogs TFV‐DP (166403‐66‐3 sodium salt), azidothymidine‐5′‐triphosphate (AZT‐TP) (B1331‐007254 triethylammonium salt), 3TC‐TP (143616‐58‐4, Triethyammonium salt), and FTC‐TP (1188407‐46‐6 triethylammonium salt) were obtained from BOC Sciences Inc (Shirley, NY).

RESTRICT master mixes contained 2.5 nM final concentration of DNA template, 500 nM of each deoxynucleotide triphosphates (dNTPs) (D7295, Sigma, St. Louis, MO), and 25 nM of 16S rRNA forward primer AGA GTT TGA TCC TGG CTC AG (51‐01‐19‐06, Integrated DNA Technologies, Coralville, IA). DNA templates had 20 nucleotide (nt) primer binding domains on their 3′ ends complementary to the 16S rRNA primer and chain terminating domains designed according to the target nucleotide analog that ranged in length from 45 to 200 nt (Table 1).

**TABLE 1:** Sequences of the chain terminating domains of DNA templates used in the RESTRICT assay

Short name	Chain terminating domain sequence
180 nt TTCA	(TTCA)_45_, that is, 45 TTCA repeats
180 nt GGCA	(GGCA)_45_
180 nt GGAA	(GGAA)_45_
135 nt TTCA	(TTCA)_33_ TTC
90 nt TTCA	(TTCA)_22_ TT
45 nt TTCA	(TTCA)_11_ T
90 nt TCAA	(TCAA)_22_ TC
90 nt TCA	(TCA)_30_
90 nt TTTCA	(TTTCA)_18_

RESTRICT assays were initiated by adding 5 μl RT (100 nM final concentration in the assay) to 35 μl of master mix in a 384‐well microplate (3575, Corning, Corning, NY), incubated at 37°C for 30 min in a microplate reader (SpectraMax iD3, Molecular Devices, San Jose, CA). Reactions were stopped by adding PicoGreen dye (P7581, ThermoFisher Scientific, Waltham, MA) diluted 1:400 in 1×TE (10128‐588, VWR, Radnor, PA) and incubating for 1 min prior to fluorescence readout. Three replicate reactions were tested unless otherwise stated.

### 3.2 Comparison between probabilistic model and experimental data

We performed a curve fit to determine *K*_aff_ values that minimized the difference between experimental and theoretical normalized fluorescence values. We completed RESTRICT assays at 500 nM using a 180 nt TTCA template, a 90 nt TCAA template, and a 180 nt GGCA template to demonstrate clinically relevant detection of TFV‐DP, AZT‐TP, and FTC‐TP, respectively. We completed RESTRICT assays at 25 nM IC using a 180 nt GGAA to measure lamivudine triphosphate (3TC‐TP). We prepared serial dilutions of each drug at concentrations ranging from 10^−11^ to 10^−5^ M in 10‐fold increments. We varied *K*_aff_ in the theoretical model from 0 to 1 and chose the value that minimized the sum of the square of the difference between experiment and theory for all data points tested for a given drug‐template pair.

We performed RESTRICT assays with the 180 nt TTCA template at dNTP concentrations of 0.10, 0.30, 1.56, and 6.25 μM using serial dilutions of TFV‐DP with concentrations ranging from 10^−10^ to 10^−5^ M in 10‐fold increments to determine the effect of nucleotide concentration on RESTRICT. We computed theoretical fluorescence intensities within the same range of dNTP concentrations using the optimal *K*_aff_ value determined at 500 nM dNTP and compared experimental and theoretical IC_50_ values.

We performed RESTRICT assays at 500 nM dNTP using the 45 nt TTCA, 90 nt TTCA, 135 nt TTCA, and 180 nt TTCA templates (Table 1) to determine the effect of template length on RESTRICT. Similarly, we performed RESTRICT assays at 500 nM dNTP using the 90 nt TCAA, 90 nt TCA, 90 nt TTCA, and 90 nt TTTCA templates to determine the effect of template sequence on RESTRICT. In both cases, we tested serial dilutions of TFV‐DP with concentrations ranging from 10^−10^ to 10^−5^ M in 10‐fold increments and compared experimental and theoretical 50% inhibition concentration (IC_50_).

### 3.3 Selective detection of nucleotide analogs

We used 180 nt GGCA DNA templates (excluding adenosine bases) for selective detection of 3TC‐TP (cytidine analog) by Watson‐Crick‐Franklin base pairing without cross‐reactivity with TFV‐DP (adenosine analog). Similarly, we used 180 nt TTCA DNA templates (excluding guanosine bases) for selective TFV‐DP detection without 3TC‐TC binding. We tested four concentrations each of TFV‐DP and 3TC‐TP between 10^−10^ and 10^−4^ M to measure inhibition with either the GGCA or TTCA template.

### 3.4 Clinical ranges for nucleotide analogs

We estimated clinical ranges, to provide guidelines of concentrations of interest for each nucleotide analog, from published liquid chromatography tandem mass spectrometry measurements. The clinical ranges for FTC‐TP, 3TC‐TP, and TFV‐DP were based on LC‐MS/MS measurements in dried blood spots (DBS) (see Table 2). We used the conversion factor of 12 fmol/3 mm DBS punch = 1 fmol/10^6^ RBCs^10^ and assumed 5 million RBCs per microliter of blood^37^ to convert from fmol/punch to molar units. Nucleotide analogs measurements in RBCs/DBS is appealing in point‐of‐care diagnostic development since RBCs are more abundant and easier to lyse than PBMCs. The clinical range for AZT‐TP was determined from peripheral blood mononuclear cells (PBMCs) because AZT‐TP does not accumulate in red blood cells.^38^

**TABLE 2:** Clinical ranges for nucleotide analogs of interest based on liquid chromatography tandem mass spectrometry measurements

Pro‐drug	Intracellular metabolite	Clinical range (M)	References
Emtricitabine (FTC)	FTC‐TP	3.83E–9 to 1.73E–7	^39^
Lamivudine (3TC)	3TC‐TP	2.50E–8 to 5.00E–7	^38^, ^40^, ^41^
Tenofovir disoproxil fumarate (TDF)	TFV‐DP	8.33E–8 to 6.25E–7	^10^, ^11^
Tenofovir alafenamide (TAF)	TFV‐DP	9.57E–10 to 6.89E–8	^39^
Zidovudine (AZT)	AZT‐TP	5.56E–9 to 5.56E–8	^42^

*Note*: The clinical ranges for FTC‐TP, 3TC‐TP, and TFV‐DP are for drug concentrations in red blood cells, while the clinical range for AZT‐TP is in peripheral blood mononuclear cells since AZT‐TP does not accumulate in red blood cells.

### 3.5 Data analysis and statistics

We normalized RESTRICT data using fluorescence from negative controls (no RT enzyme) as 0% and fluorescence from positive controls (no nucleotide analog) as 100%. We also normalized the fluorescence intensity of theoretical calculations from the probabilistic model of RT inhibition to facilitate comparison with experimental results. Fluorescence intensities from experimental data and theoretical calculations were fit to four‐parameter logistic regression curves using GraphPad Prism 9 (GraphPad Software Inc.) to obtain the 50% inhibition concentration (IC_50_).

## 4 RESULTS

### 4.1 Determination of nucleotide analog affinity coefficient

The nucleotide analog affinity coefficient, *K*_aff_, indicates the relative affinity of RT enzyme for a particular nucleotide analog compared with the endogenous nucleotide that it mimics. Given the preferences exhibited by HIV‐1 RT for different nucleotide analogs based on the nature (RNA or DNA) and sequence of nucleic acid templates,^32^, ^33^ we determined *K*_aff_ empirically for each DNA template and nucleotide analog tested. We chose Kaff values that minimized the sum of the difference between experiment and theory for all data points for each template‐drug pair. *K*_aff_ values of 0.3, 0.2, 0.2, and 0.05 provided the minimum difference between experiment and theory for TFV‐DP, FTC‐TP, AZT‐TP, and 3TC‐TP using the 180 nt TTCA, 180 nt GGCA, 90 nt TCAA, and 180 nt GGAA templates, respectively. These *K*_aff_ values were used in subsequent experiments.

### 4.2 Contributions of RESTRICT assay products to endpoint fluorescence

**FIGURE 2:**
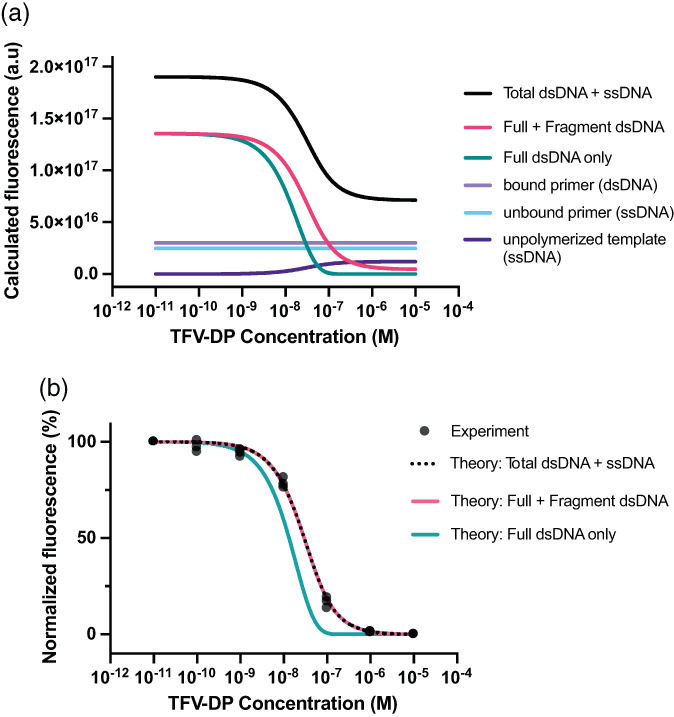
Contributions of different model components to RESTRICT assay results. (a) Fluorescence from the theoretical model for unpolymerized ssDNA, unbound primer, bound primer, full dsDNA product, as well as both full and fragment dsDNA product calculated using Equations ((1), (2), (3), (4), (5), (6), (7), (8), (9)). (b) Accounting for both full and fragment dsDNA improves the agreement between normalized theoretical and experimental data (500 nM dNTP, 180 nt TTCA template, *N* = 3)

We can account for the contributions of different assay inputs and outputs to the total and normalized fluorescence from the RESTRICT assay. Accounting for background fluorescence from primers and unpolymerized template increases the total fluorescence from the RESTRICT assay (Figure 2a). Our focus in this study was to investigate the parameters that influence the shape and position of normalized RESTRICT curves on the concentration axis rather than obtaining exact calculations of the absolute endpoint fluorescence. Consequently, we normalized fluorescence intensities to compare theoretical and experimental data (Figure 2b).

Accounting for both full and fragment dsDNA improves the agreement between experiment and theory (Figure 2b). Experimental RESTRICT data were obtained using the 180 nt TTCA template, 500 nM dNTP, and serial dilutions of TFV‐DP. When only full‐length dsDNA was included in the theoretical model, the average difference between experimental and theoretical values was 36.68 ± 19.98%. Meanwhile when both full‐length and fragment dsDNA were included, the average difference between theory and experiment decreased to 6.36 ± 4.04%. When the theoretical lines are normalized, the “Total dsDNA + ssDNA” and “Full + Fragment dsDNA” curves are identical. In other words, including background fluorescence from primers and unpolymerized template did not change the shape of the normalized RESTRICT curves or improve the agreement between normalized experimental and theoretical data. This is unsurprising since the unpolymerized ssDNA curve does not significantly impact the shape of the curve, but instead raises the fluorescence baseline of the raw data.

### 4.3 Varying dNTP concentration, template length, and template sequence

**FIGURE 3:**
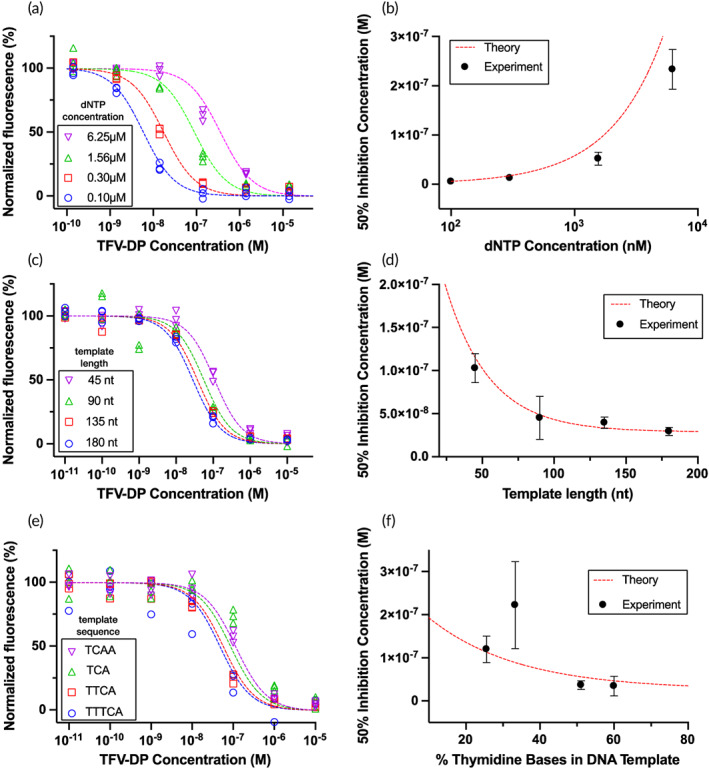
Effect of nucleotide concentration, template length, and template sequence on detection of TFV‐DP using the RESTRICT assay. (a) As dNTP concentration increases, RESTRICT curves shift to higher TFV‐DP concentrations. (b) As dNTP concentration increases, IC_50_ increases. (c) As template length increases, RESTRICT curves shift to lower TFV‐DP concentrations. (d) As template length increases, IC_50_ decreases. (e) As the thymidine base content of DNA templates increases, RESTRICT curves shift to lower TFV‐DP concentrations. (f) As thymidine content increases, IC_50_ decreases. Symbols indicate experiments and dashed lines indicate theory. *N* = 3. Error bars indicate 95% confidence intervals

We examined the effect of dNTP concentration, template length, and template sequence on RESTRICT assay performance in theory and experiment (Figure 3). As dNTP concentration increases, we observe less inhibition (and consequently higher fluorescence) as a function of TFV‐DP concentrations since higher dNTP concentrations make TFV‐DP insertion less likely and more TFV‐DP is required to have a similar inhibitory effect (Figure 3a). This has the effect of shifting the inhibition curves to the right. Figure 3b plots the IC_50_ value as an exponential function of dNTP concentration and shows good agreement between experimental data and theoretical calculations.

Figure 3c examines the effect of template length on RESTRICT by keeping dNTP concentration constant at 500 nM and varying the length of the chain terminating domain of the TTCA template. The experimental data and theory show that as template length increases, RESTRICT curves shift to the left as lower TFV‐DP concentrations now have an increased inhibitory effect, since longer DNA templates provide additional opportunities for TFV‐DP insertion. Figure 3d shows an exponential decay in IC_50_ value with increasing template length.

Figure 3e shows the effect of varying template sequence on RESTRICT inhibition curves. DNA template length was fixed at 90 nt and dNTP concentration was fixed at 500 nM, while the sequence of the chain terminating domain was varied to test the impact of thymidine content on TFV‐DP detection. Figure 3e shows that as thymidine content increases RESTRICT curves shift left towards lower TFV‐DP concentrations since there are increased opportunities for TFV‐DP incorporation (like the effect of increased template length). Figure 3f confirms this trend in both the experimental and theoretical IC_50_ values. Although the TCA template (33% thymidine content in the chain terminating domain) deviates from this trend, all the other templates tested confirmed the expected trend of lower IC_50_ as thymidine content increases given the greater number of TFV‐DP insertion sites in the chain terminating domain.

Taken together, Figure 3 shows that the probabilistic model for RESTRICT can be used to inform systematic assay design and choose appropriate assay parameters to shift RESTRICT curves to desired concentration ranges for TFV‐DP detection. The RESTRICT assay can be shifted towards more sensitive measurement of nucleotide analogs at low concentrations by decreasing the dNTP concentration, increasing the template length, and incorporating a higher fraction of nucleotides that the drug of interest will bind to during polymerization.

### 4.4 Detection of multiple nucleotide analogs

**FIGURE 4:**
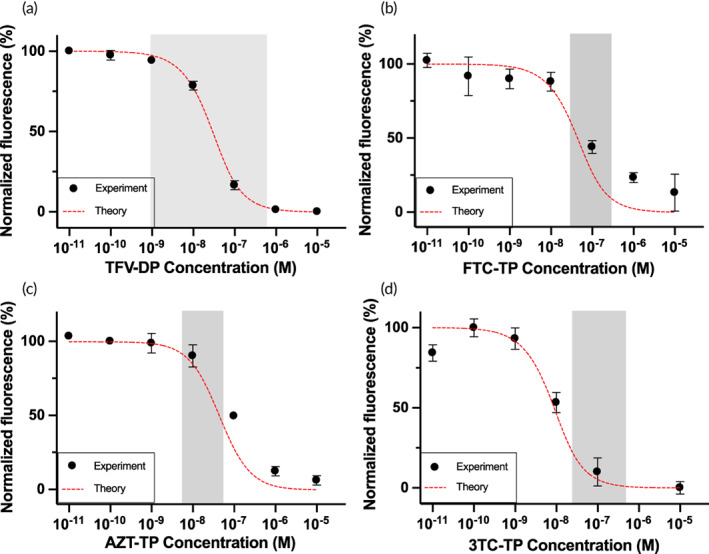
Detection of clinically relevant concentrations of multiple nucleotide analogs using RESTRICT. (a) TFV‐DP measurement using 180 nt TTCA template at 500 nM dNTP. (b) FTC‐TP measurement using 180 nt GGCA template at 500 nM dNTP. (c) AZT‐TP measurement using 90 nt TCAA template at 500 nM dNTP. (d) 3TC‐TP measurement using 180 nt GGAA template at 25 nM dNTP. Symbols indicate experiments and dashed lines indicate theory. *N* = 3. Error bars indicate standard deviation. Gray shaded regions indicate clinical range for each drug.

The RESTRICT assay accounts for the endogenous nucleotide that a drug mimics and Watson‐Crick‐Franklin base pairing between the template and the drug. We designed DNA templates to enable sensitive detection of nucleotide analogs of interest. Figure 4a shows a RESTRICT curve obtained using 500 nM dNTP, a 180 nt TTCA template, serial dilution dilutions of TFV‐DP, and an empirically derived *K*_aff_ = 0.3. Figure 4b shows a RESTRICT curve obtained using 500 nM dNTP, a 180 nt GGCA template, and serial dilutions of FTC‐TP, and empirically derived *K*_aff_ = 0.2. Figure 4c shows a RESTRICT curve obtained using 500 nM dNTP, a 90 nt TCAA template, serial dilutions of AZT‐TP, and empirically derived *K*_aff_ = 0.2. Finally, Figure 4d shows a RESTRICT curve obtained using 25 nM dNTP, a 180 nt GGAA template, serial dilutions of 3TC‐TP, and empirically derived *K*_aff_ = 0.05. Taken together, Figure 4 demonstrates that RESTRICT DNA templates can be designed to detect clinically relevant concentrations of different nucleotide analogs used in HIV treatment and prevention.

### 4.5 Selective detection of nucleotide analogs

RESTRICT can measure specific nucleotide analogs without cross‐reactivity with companion nucleotide analogs present in the same clinical sample. For example, TFV‐DP and 3TC‐TP are common companion drugs used in ART regimens.^7^ TFV‐DP in red blood cells is a measure of long‐term (1–3 month) medication adherence,^10^, ^11^ and 3TC‐TP in red blood cells indicates recent (<1 week) adherence.^41^ Selective measurement of TFV‐DP and 3TC‐TP is important to avoid conflating recent pill ingestion with long‐term adherence.

**FIGURE 5:**
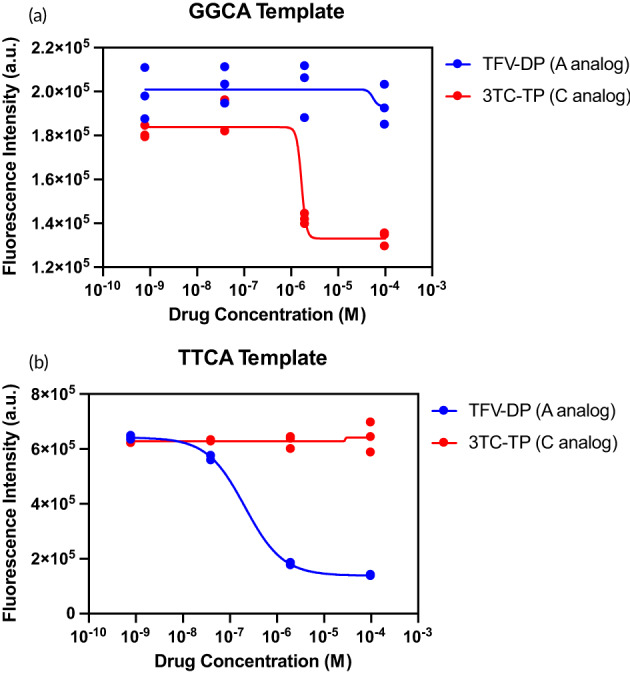
Detection of TFV‐DP and 3TC‐TP using DNA templates designed for selective detection of each drug without cross‐reactivity. TFV‐DP (A analog) was detected with a thymidine‐rich TTCA template that excluded G bases to prevent cross‐reactivity with 3TC‐TP (C analog). Similarly, 3TC‐TP was detected with a guanosine‐rich GGCA template that excluded T bases. *N* = 3

We designed a guanosine‐rich DNA template (180 nt GGAA) for selective detection of 3TC‐TP (deoxycytidine analog) and excluded thymidine bases without cross‐reactivity with TFV‐DP (deoxyadenosine analog). Similarly, we designed a thymidine‐rich DNA template that excluded guanosine bases (180 nt TTCA) for selective TFV‐DP detection without cross‐reactivity with 3TC‐TP (Figure 5). RESTRICT assays with guanosine‐rich DNA templates produced the expected inhibition with 3TC‐TP, a deoxycytidine analog but did not produce inhibition with TFV‐DP even at >1000‐fold higher than clinically relevant concentrations (see Table 2). Similarly, the thymidine‐rich TTCA template responded only to TFV‐DP and did not provide inhibition with 3TC‐TP even at concentrations >1000‐fold higher than clinically relevant concentrations.

## 5 DISCUSSION

### 5.1 Benefits of probabilistic model for RESTRICT assays

We developed a probabilistic model that guides the design and optimization of RESTRICT assay parameters including dNTP concentration, template length, and template sequence. Although other models of the inhibition of DNA synthesis by HIV‐1 RT in the presence of nucleotide analogs have previously been developed,^43^, ^44^ those models were mainly focused on *in vivo* treatment efficacy using biologically relevant HIV RNA templates. Conversely, our probabilistic model is focused on optimizing the performance of our *in vitro* diagnostic assay for measuring drug concentrations in clinical samples. The model is useful for systematic design of RESTRICT assays so that the linear region of the assay overlaps with the clinical range of the nucleotide analog of interest. To that end, we use synthetic DNA templates designed specifically designed to maximize the likelihood of RT inhibition for different nucleotide analogs. RESTRICT assays detect multiple nucleotide analogs by modifying the sequence of the template DNA and including/excluding specific nucleotides to enable Watson–Crick–Franklin base pairing of target drugs without cross reactivity with companion drugs.

### 5.2 Reproducibility and potential for quantitative testing

Our goal is to develop a test that is accurate enough to measure clinically relevant nucleotide analog concentrations in whole blood samples. Previous reports suggest that coefficient of variation (CV) less than 15% is suitable for quantitation of antiretroviral drugs in whole blood.^45^ The average CV across all our experiments in buffer was <5%, suggesting that RESTRICT assay can achieve the required precision for quantitation of clinical samples. We previously demonstrated that we could achieve a CV of 13.5% when running RESTRICT with whole blood samples diluted in water.^30^, ^31^ Improvements in sample preparation—incorporating a heating step after blood dilution to denature blood proteins (e.g., hemoglobin) that nonspecifically inhibit reverse transcriptase^46^—have improved the accuracy even further (data not shown).

### 5.3 Addressing sources of potential assay interference in clinical samples

We do not anticipate assay interference from endogenous reverse transcriptase (RT) in viremic HIV‐infected patients. Studies have estimated the RT activity for patients at very high viremia (~1,000,000 viral copies/mL) to be equivalent to <10,000 fg RT/ml.^47^ This RT activity level corresponds to ~85.5 fM of RT in undiluted plasma, which is ~7 orders of magnitude lower than the RT concentration (100 nM) required to obtain a measurable fluorescence signal after 30 min of the RESTRICT assay. Consequently, we do not anticipate measurable signals because of endogenous RT enzyme from clinical samples.

Similarly, we do not anticipate assay interference from endogenous dNTP in clinical samples. We did not observe any significant deviation from expected RESTRICT assay performance in our pilot evaluation study with 18 clinical samples.^31^ This is likely because dNTP concentrations in red blood cells and plasma are too low to effectively compete with nucleotide analogs for incorporation into complementary DNA strands synthesized by HIV RT. Our inclusion of a 10‐fold blood dilution step—to reduce non‐specific enzyme inhibition by blood components—further reduces these already low dNTP concentrations. Although dNTP concentrations have been measured in white blood cells including among people receiving HIV medication,^48^ we are not aware of any reports of the concentrations of dNTPs in plasma or red blood cells. Nevertheless, our pilot data with clinical samples suggest that dNTP concentrations in clinical samples do not pose a significant source of variation in the assay.

### 5.4 Potential for integration into point‐of‐care format

RESTRICT assays are completed in less than 1 h using readily available nucleic acid analysis reagents and a fluorescence reader.^30^ RESTRICT assays are user‐friendly and could be integrated into a POC format for use directly at the point of need (e.g., patient's home, doctor's office, or event setting) by using freeze‐dried reagents and a low‐cost fluorescence reader. DNA amplification reactions that use similar nucleic acid analysis reagents to RESTRICT (i.e., reverse transcriptase, primers, DNA templates, and nucleotides) have been previously integrated into POC formats by our group and others.^49^, ^50^, ^51^ We have previously shown that blood dilution to lyse red blood cells is a simple and effective sample preparation strategy to release TFV‐DP from red blood cells and minimize non‐specific RT inhibition by blood matrix components in whole blood samples from PrEP clients.^31^ RESTRICT requires <1 μl of whole blood per test. Each reaction requires addition of 5 μl of 10% whole blood (diluted in water) to 35 μl of reaction mix.

### 5.5 Deviations between theoretical and experimental RESTRICT

The goal of the theoretical framework is to predict the trends of RESTRICT's fluorescence output and IC_50_ as a function of the independent assay design parameters: dNTP concentration, template composition, and length. In this way, the theory can be used to optimize RESTRICT assays by guiding users on how to achieve a desired performance by modifying the template and reaction concentrations. Figure 3 shows that the model can predict the fluorescence and IC50 trends as a function of dNTP concentration, template composition, and length.

The variation in Figure 3b—where there appears to be a growing difference between theory and experiment as dNTP concentration increased—could indicate systematic deviations. For the 1.56 and 6.25 μM data points, experimental data undershoots the theoretical data. This could suggest that at these higher dNTP concentrations (and correspondingly higher template concentrations), additional time might be required to synthesize dsDNA using all available ssDNA templates. We used the same RT enzyme concentration (100 nM) and reaction incubation time (30 min) for all four dNTP concentrations. Additional assay optimization could ensure that the template is fully polymerized, as predicted in the model, and improve the agreement between the model and experiment.

Figure 3f shows good agreement between the model and experiments for all the experimental data except for 33.3% (TCA). We repeated this experiment on multiple occasions and obtained similar results. Since each experiment in Figure 3f provided in this figure is performed on a different template, we hypothesize that the deviation is due to errors in manufacturing the 33.3% template. Alternatively, there have been reports that HIV RT can exhibit DNA sequence preferences.^32^, ^33^ Overall, the model predicts the overall trends of RESTRICT fluorescence and $IC_{50}$ as a function of the assay parameters and is effective in aiding in the design of RESTRICT assays for measuring specific nucleotide analogs in a particular clinically relevant concentration range.

### 5.6 Limitations of RESTRICT assay

RESTRICT currently focuses on nucleotide analogs (like TFV‐DP, FTC‐TP, and 3TC‐TP) that accumulate in RBCs which can be lysed for release of the drug by blood dilution in water. Extending RESTRICT to drugs that accumulate primarily in PBMCs rather than RBCs (e.g., AZT‐TP) would require additional sample preparation to isolate and lyse PBMCs. This work shows that the model can predict RESTRICT's fluorescence and IC50 trends as a function of the dNTP concentration as well as the template length and composition. The empirically derived *K*_aff_ depends on the type (DNA or RNA) and sequence of nucleic acid template, and the choice of RT enzyme used. If the intent is to use the model to quantitatively predict RESTRICT's output fluorescence, *K*_aff_ needs to be determined empirically for each template‐drug pair.

## 6 CONCLUSIONS

Our results demonstrate that RESTRICT assays can detect multiple nucleotide analog drugs used in HIV treatment and prevention. The activity‐based approach to measuring nucleotide analogs in clinical samples presented here could be extended to detect other drugs used in infectious and noncommunicable disease management. Nucleotide analogs and polymerase inhibitors are used to treat hepatitis B,^52^ herpes,^53^ tuberculosis,^54^ cancer,^55^, ^56^ and COVID‐19.^57^ RESTRICT may support therapeutic monitoring and precision dosing of nucleotide analogs for these diseases to ensure efficacy and safety.

## AUTHOR CONTRIBUTIONS

**Ayokunle O. Olanrewaju:** Conceptualization (lead); data curation (lead); formal analysis (lead); funding acquisition (supporting); investigation (lead); methodology (lead); project administration (supporting); software (lead); supervision (supporting); validation (lead); visualization (lead); writing – original draft (lead); writing – review and editing (lead). **Benjamin P. Sullivan:** Conceptualization (supporting); formal analysis (supporting); investigation (supporting); methodology (supporting); software (lead); validation (supporting); visualization (supporting); writing – original draft (supporting); writing – review and editing (supporting). **Alicia H. Gim:** Data curation (supporting); formal analysis (supporting); investigation (supporting); methodology (supporting); software (supporting); validation (supporting); visualization (supporting). **Cosette A. Craig:** Data curation (supporting); formal analysis (supporting); investigation (supporting); methodology (supporting); visualization (supporting). **Derin Sevenler:** Conceptualization (supporting); formal analysis (supporting); investigation (supporting); methodology (supporting); visualization (supporting); writing – review and editing (supporting). **Andrew T. Bender:** Conceptualization (supporting); investigation (supporting); methodology (supporting); visualization (supporting); writing – review and editing (supporting). **Paul K. Drain:** Conceptualization (supporting); formal analysis (supporting); funding acquisition (lead); investigation (supporting); project administration (supporting); resources (lead); supervision (supporting); validation (supporting); visualization (supporting); writing – review and editing (supporting). **Jonathan D. Posner:** Conceptualization (supporting); data curation (supporting); formal analysis (supporting); funding acquisition (lead); investigation (supporting); methodology (supporting); project administration (lead); resources (lead); supervision (lead); validation (supporting); visualization (supporting); writing – original draft (supporting); writing – review and editing (supporting).

## FUNDING INFORMATION

This work was funded by National Institutes of Health (R01AI157756, R01AI136648, R21AI127200), University of Washington CoMotion Innovation Gap Fund Award, Mistletoe Research Fellowship. This work was supported by the Atlanta Center for Microsystems Engineered Point‐of‐Care Technologies funded by the National Institute of Biomedical Imaging and Bioengineering of the National Institutes of Health under Award Number U54EB027690. The content is solely the responsibility of the authors and does not necessarily represent the official views of the National Institutes of Health. This work was conducted using equipment in the Biochemical Diagnostics Foundry for Translational Research supported by the M. J. Murdock Charitable Trust. Research reported in this publication was supported by the University of Washington/Fred Hutch Center for AIDS Research, an NIH‐funded program under award number AI027757 which is supported by the following NIH Institutes and Centers: NIAID, NCI, NIMH, NIDA, NICHD, NHLBI, NIA, NIGMS, and NIDDK.

## CONFLICT OF INTEREST

Ayokunle O. Olanrewaju, Benjamin P. Sullivan, Derin Sevenler, Andrew T. Bender, Paul K. Drain, and Jonathan D. Posner are listed as inventors on a patent filed based on this work (PCT/US2020/037609).

### PEER REVIEW

The peer review history for this article is available at https://publons.com/publon/10.1002/btm2.10369.

## Data Availability

All data and code used in the analyses are available at: doi.org/10.5281/zenodo.5140303

## References

[1] UNAIDS . UNAIDS Data 2019 ; 2019: 1‐476. https://www.unaids.org/en/resources/documents/2019/2019-UNAIDS-data Accessed August 6, 2021.

[2] Amico KR , Stirratt MJ . Adherence to preexposure prophylaxis: current, emerging, and anticipated bases of evidence. Clin Infect Dis. 2014;59(suppl_1):S55‐S60. doi:10.1093/cid/ciu266 24926036 PMC4060253

[3] Drain PK , Bardon AR , Simoni JM , et al. Point‐of‐care and near real‐time testing for antiretroviral adherence monitoring to HIV treatment and prevention. Curr HIV/AIDS Rep. 2020;17(5):487‐498. doi:10.1007/s11904-020-00512-3 32627120 PMC7492442

[4] Castillo‐Mancilla JR , Morrow M , Coyle RP , et al. Tenofovir diphosphate in dried blood spots is strongly associated with viral suppression in individuals with human immuno‐deficiency virus infections. Clin Infect Dis. 2019;68(8):1335‐1342. doi:10.1093/cid/ciy708 30137238 PMC6451996

[5] Castillo‐Mancilla JR , Haberer JE . Adherence measurements in HIV: new advancements in pharmacologic methods and real‐time monitoring. Curr HIV/AIDS Rep. 2018;15(1):49‐59. doi:10.1007/s11904-018-0377-0 29380227 PMC5876155

[6] Dimitrov DT , Mâsse BR , Donnell D . PrEP adherence patterns strongly impact individual HIV risk and observed efficacy in randomized clinical trials. J Acquir Immune Defic Syndr. 2016;72(4):444‐451. doi:10.1097/QAI.0000000000000993 26990823 PMC4925182

[7] Saag MS , Gandhi RT , Hoy JF , et al. Antiretroviral drugs for treatment and prevention of HIV infection in adults: 2020 recommendations of the international antiviral society–USA panel. JAMA. 2020;324(16):1651‐1669. doi:10.1001/jama.2020.17025 33052386 PMC11017368

[8] Anderson PL , Kakuda TN , Lichtenstein KA . The cellular pharmacology of nucleoside‐ and nucleotide‐analogue reverse‐transcriptase inhibitors and its relationship to clinical toxicities. Clin Infect Dis. 2004;38(5):743‐753. doi:10.1086/381678 14986261

[9] World Health Organization (WHO) . Policy Brief: WHO Expands Recommendation on Oral Pre‐Exposure Prophylaxis of HIV Infection (PrEP). World Health Organization (WHO); 2015. http://www.who.int/hiv/pub/prep/policy-brief-prep-2015/en/ Accessed July 26, 2019.

[10] Castillo‐Mancilla JR , Zheng JH , Rower JE , et al. Tenofovir, emtricitabine, and tenofovir diphosphate in dried blood spots for determining recent and cumulative drug exposure. AIDS Res Hum Retroviruses. 2013;29(2):384‐390. doi:10.1089/aid.2012.0089 22935078 PMC3552442

[11] Anderson PL , Liu AY , Castillo‐Mancilla JR , et al. Intracellular tenofovir‐diphosphate and emtricitabine‐triphosphate in dried blood spots following directly observed therapy. Antimicrob Agents Chemother. 2018;62(1):e01710‐17. doi:10.1128/AAC.01710-17 29038282 PMC5740314

[12] Kearney BP , Flaherty JF , Shah J . Tenofovir disoproxil fumarate: clinical pharmacology and pharmacokinetics. Clin Pharmacokinet. 2004;43(9):595‐612. doi:10.2165/00003088-200443090-00003 15217303

[13] Castillo‐Mancilla J , Seifert S , Campbell K , et al. Emtricitabine‐triphosphate in dried blood spots as a marker of recent dosing. Antimicrob Agents Chemother. 2016;60(11):6692‐6697. doi:10.1128/AAC.01017-16 27572401 PMC5075074

[14] Ibrahim ME , Castillo‐Mancilla JR , Yager J , et al. Individualized adherence benchmarks for HIV pre‐exposure prophylaxis. AIDS Res Hum Retroviruses. 2020;37(6):421‐428. doi:10.1089/AID.2020.0108 33191774 PMC8213008

[15] Morrow M , MaWhinney S , Coyle RP , et al. Predictive value of tenofovir diphosphate in dried blood spots for future viremia in persons living with HIV. J Infect Dis. 2019;220(4):635‐642. doi:10.1093/infdis/jiz144 30942881 PMC6639595

[16] Spinelli MA , Glidden DV , Anderson PL , et al. Brief report: Short‐term adherence marker to PrEP predicts future nonretention in a large PrEP demo project: implications for point‐of‐care adherence testing. J Acquir Immune Defic Syndr. 2019;81(2):158‐162. doi:10.1097/QAI.0000000000002005 31095005 PMC6530484

[17] Coulier L , Gerritsen H , van Kampen JJA , et al. Comprehensive analysis of the intracellular metabolism of antiretroviral nucleosides and nucleotides using liquid chromatography–tandem mass spectrometry and method improvement by using ultra performance liquid chromatography. J Chromatogr B. 2011;879(26):2772‐2782. doi:10.1016/j.jchromb.2011.07.045 21862423

[18] Jansen RS , Rosing H , Schellens JHM , Beijnen JH . Mass spectrometry in the quantitative analysis of therapeutic intracellular nucleotide analogs. Mass Spectrom Rev. 2011;30(2):321‐343. doi:10.1002/mas.20280 20623700

[19] Celum CL , Mgodi N , Bekker LG , et al. PrEP adherence and effect of drug level feedback among young African women in HPTN 082. Paper presented at Proceedings of the 10th International AIDS Society Meeting, Mexico City, Mexico:30‐31; 2019.

[20] Pu F , Zhang W , Bateman KP , Liu Y , Helmy R , Ouyang Z . Using miniature MS system with automatic blood sampler for preclinical pharmacokinetics study. Bioanalysis. 2017;9(21):1633‐1641. doi:10.4155/bio-2017-0160 29095035 PMC5771462

[21] Pu F , Pandey S , Bushman LR , Anderson PL , Ouyang Z , Cooks RG . Direct quantitation of tenofovir diphosphate in human blood with mass spectrometry for adherence monitoring. Anal Bioanal Chem. 2020;412(6):1243‐1249. doi:10.1007/s00216-019-02304-0 31897555 PMC8439565

[22] Li L , Chen TC , Ren Y , Hendricks PI , Cooks RG , Ouyang Z . Mini 12, miniature mass spectrometer for clinical and other applications—introduction and characterization. Anal Chem. 2014;86(6):2909‐2916. doi:10.1021/ac403766c 24521423 PMC3985695

[23] Bardon AR , Simoni JM , Layman LM , Stekler JD , Drain PK . Perspectives on the utility and interest in a point‐of‐care urine tenofovir test for adherence to HIV pre‐exposure prophylaxis and antiretroviral therapy: an exploratory qualitative assessment among U.S. clients and providers. AIDS Res Therapy. 2020;17(1):50. doi:10.1186/s12981-020-00308-w PMC7412814 32762713

[24] Drain P , Ngure K , Mugo N , et al. Testing a real‐time tenofovir urine adherence assay for monitoring and providing feedback to preexposure prophylaxis in Kenya (PUMA): protocol for a pilot randomized controlled trial. JMIR Res Protoc. 2020;9(4):e15029. doi:10.2196/15029 32238341 PMC7163413

[25] Gandhi M , Bacchetti P , Rodrigues WC , et al. Development and validation of an immunoassay for tenofovir in urine as a real‐time metric of antiretroviral adherence. EClinicalMedicine. 2018;2–3:22‐28. doi:10.1016/j.eclinm.2018.08.004 PMC6428441 30906930

[26] Gandhi MM , Bacchetti P , Spinelli MAM , et al. Validation of a urine tenofovir immunoassay for adherence monitoring to PrEP and ART and establishing the cutoff for a point‐of‐care test. J Acquir Immune Defic Syndr. 2019;81(1):72‐77. doi:10.1097/QAI.0000000000001971 30664078 PMC6456396

[27] Spinelli MA , Glidden DV , Rodrigues WC , et al. Low tenofovir level in urine by a novel immunoassay is associated with seroconversion in a preexposure prophylaxis demonstration project. AIDS. 2019;33(5):867‐872. doi:10.1097/QAD.0000000000002135 30649051 PMC6375797

[28] Sevenler D , Bardon A , Fernandez Suarez M , et al. Immunoassay for HIV drug metabolites tenofovir and tenofovir diphosphate. ACS Infect Dis. 2020;6(7):1635‐1642. doi:10.1021/acsinfecdis.0c00010 32392030 PMC7895468

[29] Spinelli MA , Haberer JE , Chai PR , Castillo‐Mancilla J , Anderson PL , Gandhi M . Approaches to objectively measure antiretroviral medication adherence and drive adherence interventions. Curr HIV/AIDS Rep. 2020;17(4):301‐314. doi:10.1007/s11904-020-00502-5 32424549 PMC7363551

[30] Olanrewaju AO , Sullivan BP , Zhang JY , et al. Enzymatic assay for rapid measurement of antiretroviral drug levels. ACS Sens. 2020;5(4):952‐959. doi:10.1021/acssensors.9b02198 32248685 PMC7183420

[31] Olanrewaju AO , Sullivan BP , Bardon AR , et al. Pilot evaluation of an enzymatic assay for rapid measurement of antiretroviral drug concentrations. Virol J. 2021;18(1):77. doi:10.1186/s12985-021-01543-x 33858461 PMC8048217

[32] Huber HE , McCoy JM , Seehra JS , Richardson CC . Human immunodeficiency virus 1 reverse transcriptase. Template binding, processivity, strand displacement synthesis, and template switching. J Biol Chem. 1989;264(8):4669‐4678. 2466838

[33] Starnes MC , Cheng YC . Human immunodeficiency virus reverse transcriptase‐associated RNase H activity. J Biol Chem. 1989;264(12):7073‐7077. 2468665

[34] Dragan AI , Casas‐Finet JR , Bishop ES , Strouse RJ , Schenerman MA , Geddes CD . Characterization of picoGreen interaction with dsDNA and the origin of its fluorescence enhancement upon binding. Biophys J. 2010;99(9):3010‐3019. doi:10.1016/j.bpj.2010.09.012 21044599 PMC2965993

[35] Singer VL , Jones LJ , Yue ST , Haugland RP . Characterization of picoGreen reagent and development of a fluorescence‐based solution assay for double‐stranded DNA quantitation. Anal Biochem. 1997;249(2):228‐238. doi:10.1006/abio.1997.2177 9212875

[36] Le Grice SFJ , Cameron CE , Benkovic SJ . [13] Purification and characterization of human immunodeficiency virus type 1 reverse transcriptase. Methods in Enzymology. Vol 262. DNA Replication. Academic Press; 1995:130‐144. doi:10.1016/0076-6879(95)62015-X 8594344

[37] Dean L. Blood and the Cells It contains. National Center for Biotechnology Information (US); 2005. https://www.ncbi.nlm.nih.gov/books/NBK2263/ Accessed June 15, 2022.

[38] Durand‐Gasselin L , Silva DD , Benech H , Pruvost A , Grassi J . Evidence and possible consequences of the phosphorylation of nucleoside reverse transcriptase inhibitors in human red blood cells. Antimicrob Agents Chemother. 2007;51(6):2105‐2111. doi:10.1128/AAC.00831-06 17438052 PMC1891370

[39] Yager J , Castillo‐Mancilla J , Ibrahim ME , et al. Intracellular tenofovir‐diphosphate and emtricitabine‐triphosphate in dried blood spots following tenofovir alafenamide: the TAF‐DBS study. J Acquir Immune Defic Syndr. 2020;84(3):323‐330. doi:10.1097/QAI.0000000000002354 32539288

[40] Adams JL , Sykes C , Menezes P , et al. Tenofovir diphosphate and emtricitabine triphosphate concentrations in blood cells compared with isolated peripheral blood mononuclear cells: a new measure of antiretroviral adherence? J Acquir Immune Defic Syndr. 2013;62(3):260‐266. doi:10.1097/QAI.0b013e3182794723 23111578 PMC4042836

[41] Lofgren SM , Nicol MR , Kandole TK , et al. Short communication: A descriptive analysis of dried blood spot adherence testing among Ugandans with HIV presenting with cryptococcal meningitis. AIDS Res Hum Retroviruses. 2021;37(7):529‐533. doi:10.1089/aid.2020.0202 33677986 PMC8260886

[42] Anderson PL , Zheng JH , King T , et al. Concentrations of zidovudine‐ and lamivudine‐triphosphate according to cell type in HIV‐seronegative adults. AIDS. 2007;21(14):1849‐1854. doi:10.1097/QAD.0b013e3282741feb 17721092

[43] Duwal S , von Kleist M . Top‐down and bottom‐up modeling in system pharmacology to understand clinical efficacy: an example with NRTIs of HIV‐1. Eur J Pharm Sci. 2016;94:72‐83. doi:10.1016/j.ejps.2016.01.016 26796142

[44] Duwal S , Dickinson L , Khoo S , von Kleist M . Hybrid stochastic framework predicts efficacy of prophylaxis against HIV: an example with different dolutegravir prophylaxis schemes. PLoS Comput Biol. 2018;14(6):e1006155. doi:10.1371/journal.pcbi.1006155 29902179 PMC6001963

[45] Pandey S , Hu Y , Bushman LR , Castillo‐Mancilla J , Anderson PL , Cooks RG . Miniature mass spectrometer‐based point‐of‐care assay for cabotegravir and rilpivirine in whole blood. Anal Bioanal Chem. 2022;15:3387‐3395. doi:10.1007/s00216-022-03954-3 PMC9018536 35169905

[46] Sidstedt M , Hedman J , Romsos EL , et al. Inhibition mechanisms of hemoglobin, immunoglobulin G, and whole blood in digital and real‐time PCR. Anal Bioanal Chem. 2018;410(10):2569‐2583. doi:10.1007/s00216-018-0931-z 29504082 PMC5857286

[47] Malmsten A , Shao XW , Aperia K , et al. HIV‐1 viral load determination based on reverse transcriptase activity recovered from human plasma. J Med Virol. 2003;71(3):347‐359. doi:10.1002/jmv.10492 12966539

[48] Chen X , Castillo‐Mancilla JR , Seifert SM , et al. Analysis of the endogenous deoxynucleoside triphosphate pool in HIV‐positive and ‐negative individuals receiving tenofovir‐emtricitabine. Antimicrob Agents Chemother. 2016;60(9):5387‐5392. doi:10.1128/AAC.01019-16 27353267 PMC4997838

[49] Bender AT , Borysiak MD , Levenson AM , Lillis L , Boyle DS , Posner JD . Semiquantitative nucleic acid test with simultaneous isotachophoretic extraction and amplification. Anal Chem. 2018;90(12):7221‐7229. doi:10.1021/acs.analchem.8b00185 29761701 PMC6504979

[50] Lafleur LK , Bishop JD , Heiniger EK , et al. A rapid, instrument‐free, sample‐to‐result nucleic acid amplification test. Lab Chip. 2016;16(19):3777‐3787. doi:10.1039/C6LC00677A 27549897

[51] Natoli ME , Rohrman BA , De Santiago C , van Zyl GU , Richards‐Kortum RR . Paper‐based detection of HIV‐1 drug resistance using isothermal amplification and an oligonucleotide ligation assay. Anal Biochem. 2018;544:64‐71. doi:10.1016/j.ab.2017.12.008 29229373 PMC5854266

[52] Manolakopoulos S , Striki A , Papastergiou V , et al. Persistence and adherence to nucleos(t)ide analogues in chronic hepatitis B: a multicenter cohort study. Eur J Gastroenterol Hepatol. 2020;32(5):635‐641. doi:10.1097/MEG.0000000000001558 31688309

[53] Cies JJ , Moore WS , Miller K , et al. Therapeutic drug monitoring of continuous‐infusion acylovir for disseminated herpes simplex virus infection in a neonate receiving concurrent extracorporeal life support and continuous renal replacement therapy. Pharmacotherapy. 2015;35(2):229‐233. doi:10.1002/phar.1526 25556960

[54] MGG S , Mulder LW , de Jager A , et al. Pharmacokinetic modeling and optimal sampling strategies for therapeutic drug monitoring of rifampin in patients with tuberculosis. Antimicrob Agents Chemother. 2015;59(8):4907‐4913. doi:10.1128/AAC.00756-15 26055359 PMC4505200

[55] Cohen S , Jordheim LP , Megherbi M , Dumontet C , Guitton J . Liquid chromatographic methods for the determination of endogenous nucleotides and nucleotide analogs used in cancer therapy: a review. J Chromatogr B. 2010;878(22):1912‐1928. doi:10.1016/j.jchromb.2010.05.016 20558114

[56] Jordheim LP , Durantel D , Zoulim F , Dumontet C . Advances in the development of nucleoside and nucleotide analogues for cancer and viral diseases. Nat Rev Drug Discov. 2013;12(6):447‐464. doi:10.1038/nrd4010 23722347

[57] Chien M , Anderson TK , Jockusch S , et al. Nucleotide analogues as inhibitors of SARS‐CoV‐2 polymerase, a key drug target for COVID‐19. J Proteome Res. 2020;19(11):4690‐4697. doi:10.1021/acs.jproteome.0c00392 32692185 PMC7640960

